# Characterization of suicidal depression: A 1 year prospective study

**DOI:** 10.1192/j.eurpsy.2022.16

**Published:** 2022-04-18

**Authors:** Bénédicte Nobile, Emilie Olié, Jonathan Dubois, Myriam Benramdane, Sébastien Guillaume, Philippe Courtet

**Affiliations:** 1 Department of Emergency Psychiatry and Acute Care, Lapeyronie Hospital, CHU Montpellier, Montpellier, France; 2 IGF, CNRS, INSERM, University of Montpellier, Montpellier, France; 3 FondaMental Foundation, Montpellier, France

**Keywords:** Depression, inpatients, suicidal ideation, suicide attempt

## Abstract

**Background:**

Suicidal ideation (SI) is an important risk factor of death by suicide. Recent data suggest that suicidal depression (i.e., moderate to severe depression with SI) could be a specific depression subtype with worse clinical outcomes than nonsuicidal depression (i.e., without SI).

**Methods:**

Among 898 French adult inpatients (67% women, mean age: 41.23 [SD: 14.33]) with unipolar depression, 71.94% had moderate to severe depression (defined using the cut-offs of validated scales: beck depression inventory, clinician-rated 30-item inventory depression symptomatology, and quick inventory of depressive symptomatology) and among them, 63.6% had SI according to the suicidal item (score ≥ 2) of the depression scale they filled in. Clinical features (anxiety, psychological pain, and hopelessness) were assessed at baseline. The occurrence of a suicide attempt (SA) or a suicide event (SE) (i.e., actual, aborted or interrupted SA, or hospitalization for SI) was recorded during the 1-year follow-up. The risk of actual SA and SE was compared between groups with adjusted Cox regression models.

**Results:**

The risk of actual SA and SE during the follow-up was 2- and 1.8-fold higher, respectively, in patients with suicidal depression, independently of potential cofounders such as history of lifetime SA, age, sex, and baseline depression severity.

**Conclusions:**

Suicidal depression is associated with poorer prognosis in terms of actual SA/SE, despite optimal care (i.e., care in a hospital department specialized in the management of suicidal crisis). Specific therapeutic strategies might be needed for these patients.

## Introduction

Suicidal behaviors (i.e., death by suicide and suicide attempts [SA, actual, aborted, or interrupted]) represent a major public health problem. About 800,000 people die by suicide worldwide each year, and there are 20–30 times more suicide attempters [1,2]. Although suicidal ideation (SI), especially active SI (i.e. thought of suicide with plan), can be considered the first step of a continuum (from SI to SA and to death by suicide) [3], clinical practice focuses more on previous SA to prevent recurrence rather than on SI [4]. Two meta-analyses found that SI is the third most potent risk factor for future death by suicide (following lifetime history of SA and prior psychiatric hospitalization) [5], and that previous SA is not more strongly associated with death by suicide than SI [6]. Yet, SI is still considered a symptom or a consequence of a concomitant psychiatric disorder, mainly major depressive disorder [7]. Therefore, many clinicians think that by treating the psychiatric disorder, SI also will disappear [3]. Consequently, nonspecific anti-suicidal treatments [8], particularly antidepressants [7], are often used in patients with depression and SI. Treatment with antidepressants has been associated with a decrease in suicidal risk, but it is not considered sufficient [9]. Indeed, the American Psychological Association clinical practice guidelines state that: “Evidence for a lowering of suicide rates with antidepressant treatment is inconclusive” [10]. Furthermore, suicidal patients (with lifetime history of SA and/or current SI) respond less to this drug class [11,12]. Specific pharmacological treatments (e.g., ketamine and buprenorphine) [13,14] and psychotherapies (e.g., acceptance and commitment therapy) [15] are showing promising results on suicidal risk. Similarly, suicide-focused cognitive behavioral therapy efficiently reduces SA and/or SI [16], but not depression-focused psychotherapies [3,17,18]. This suggests that moderate to severe depression with active SI (i.e., suicidal depression) could be a specific subtype of depression with its own pathophysiology, clinical features, and management [19,20].

Indeed, patients with suicidal depression have more severe baseline clinical features, respond less well to antidepressants, may have a different clinical course (e.g., depression remission) and have higher suicidal risk than depressed patients without SI [21–25]. We previously reported similar results in a study on two large cohorts of outpatients with unipolar depression [24]. Moreover, up to 20% of patients with SI report persistent SI despite depression remission [24,26]. A factor analysis of depressive syndrome showed that latent variable structures are significantly different in depressed patients with and without SI [27]. Finally, depression symptoms can vary among patients, resulting in a heterogeneous clinical picture [28]. These data support the hypothesis of major differences between suicidal and nonsuicidal patients with depression.

Therefore, we decided to better characterize suicidal depression in inpatients with unipolar depression. Even if suicidal behaviors occur across the spectrum of psychiatric disorders and can be seen as a transdiagnostic phenotype, we focused on patients with unipolar depression for three main reasons. Firstly, the aim of this study was to ask whether suicidal unipolar depression could be a specific phenotype. Secondly, this is a longitudinal study and bipolar or schizophrenic depression do not have the same clinical course as unipolar depression, and this could influence the risk of SA. For example, patients with bipolar disorder and mixed features are at higher risk of SA than patients with bipolar disorder and mania [29]. Finally, the treatments used in these disorders are different and this also may modulate the risk of SA. Specifically, lithium salts, which are often used in mood disorders, can also reduce SI and SA [30]. Clozapine used in schizophrenia is the only treatment with an authorization of use in schizophrenic suicidal patients [31]. Large cohorts would be needed to adjust for these potential confounders and/or to do sensitivity analyses.

The main objectives of this naturalistic study were: (a) to clinically characterize inpatients with moderate to severe unipolar depression and current SI (i.e., suicidal depression) at admission compared with patients with nonsuicidal depression; and (b) to investigate their risk of actual SA and Suicidal Event (SE) (i.e., actual SA, aborted SA, interrupted SA, and hospitalization for SI) during a 1-year follow-up.

## Methods

### Participants’ recruitment

This observational, prospective, naturalistic study concerned a cohort of 898 adult inpatients admitted to the Department of Emergency Psychiatry and Acute Care of Montpellier Academic Hospital, France, with the main diagnosis of unipolar major depressive episode according to the DSM-5 criteria.

Inclusion criteria were: older than 18 years of age, hospitalization at our Department in the Academic Hospital of Montpellier (France), main diagnosis of major depressive episode according to the DSM-5 criteria, capacity to speak and understand French.

This study was performed according to the French regulatory guidelines and current codes of Good Clinical Practice. Each patient was informed about the study aims and procedures and signed a written informed consent. The study protocol was approved by a local independent ethics committee (IRB-MTP_2012_07_202100867).

### Clinical assessment

This naturalistic study did not interfere with the clinical management of the included patients. Consequently, all questionnaires were not filled in by all patients at all follow-up visits due to lack of time or patient limitations (mood state, lack of energy, and unavailability).

Clinicians recorded sociodemographic data (i.e., age, sex, education level, professional activity, marital status, and having children), history of lifetime SA, number of lifetime SA, age at first SA, and current psychotropic treatments (e.g., antipsychotics, antidepressants, and anxiolytics) at admission.

The baseline clinical assessment was carried out few days after hospitalization and was based on interviews performed by trained psychiatrists or trained psychologists and self-questionnaires. All tools used to assess patients were validated for daily practice and clinical research. The diagnosis of unipolar depression was done with the Mini International Neuropsychiatric Interview (MINI-5) [32] and confirmed by experienced psychiatrists.

Psychopathology and depression were evaluated in all patients as follows:

1. Psychopathology using the MINI-5 [32].
2. Depression using the clinician-rated 30-item Inventory Depression Symptomatology (IDSC30), and/or the self-rated Quick Inventory of Depressive Symptomatology (QIDS), and/or the self-rated Beck Depression Inventory (BDI). High scores indicate high depression severity [33,34]. The choice of the depression scale was based on the clinician’s preference.

Optional assessments were (the number of concerned patients is reported in Table 1):

1. Characteristics of the last SA using the Risk/Rescue Rating Scale (RRRS) [35] and Suicidal Intent Scale (SIS) [36]. The RRRS assesses the SA lethality, defined as the probability of inflicting irreversible damage. This scale includes ten items (scored 1, 2, or 3): five items describe risk factors (risk score) and five items describe rescue factors (rescue score). High RRRS scores indicate high SA lethality. The SIS includes 15 items that are scored from 0 to 2 to define the attempt severity. The SIS comprises two parts: objective circumstances of the SA (planning subscale), and the patient’s self-reported intentions and expectations regarding the SA (conceptualization subscale). High SIS scores indicate high intent to die [37].
2. Current SI with the 19-item Beck Scale for Suicide Ideation (BSSI). Each item is scored from 0 to 2 in ascending order of severity. High scores indicate high SI intensity [38].
3. Impulsivity using the Barratt Impulsiveness Scale (BIS) [39]. Higher scores indicate higher impulsivity.
4. Hopelessness with the Beck Hopelessness Scale (BHS) [40]. Higher scores indicate higher feeling of hopelessness.
5. Reasons for Living Inventory (RFLI) [41]. Higher scores indicate higher reasons for living.
6. Emotional lability using the Affective Intensity Measure (AIM) and Affective Lability Scale (ALS) [42,43]. Higher scores indicate higher emotional lability.
7. Anxiety using the State/Trait Anxiety Inventory (STAI) [44]. Higher scores indicate higher anxiety.
8. History of childhood trauma using the Childhood Trauma Questionnaire (CTQ) [45]. The CTQ explores five dimensions of childhood trauma: physical abuse, physical neglect, emotional abuse, emotional neglect, and sexual abuse. The cut-off scores for the different subscales are ≥10 for physical abuse; ≥8 for sexual abuse; ≥13 for emotional abuse; ≥15 for emotional neglect; and ≥10 for physical neglect.
9. Level of psychological pain, physical pain, and SI using visual analog scales (VAS) for current, maximum, and usual pain/SI in the last 15 days. Usual pain/SI defines the average level during the last 15 days, whereas maximum pain/SI defines the maximum level in the last 15 days. These VAS range from 0 to 10 (from no pain/SI to highest pain/SI) [46].
10. Feelings of loneliness using the French version (ESUL) of the University of California, Los Angeles Loneliness Scale. This self-questionnaire includes 20 items to quantify the severity of the feeling of loneliness [47].
11. Therapeutic observance was assessed with the 10-item Medication Adherence Report Scale (MARS). High scores indicate good adherence [48].

**Table 1.** Comparison of baseline characteristics in patients with suicidal depression and patients with nonsuicidal depression.

Variables	Suicidal depression									
	No		Yes							
	*N* = 239		*N* = 407		Model 0		Model 1		Model 2	
	Mean (SD)/*N* (%)		Mean (SD)/*N* (%)		OR [95% CI]	*p-*value	OR [95% CI]	*p-*value	OR [95% CI]	*p-*value
Sex						**0.04**		**0.02**		**0.002**
Women	176	73.6	268	65.8	1		1		1	
Men	63	26.4	139	34.2	1.45 [1.02; 2.06]		1.56 [1.08; 2.25]		1.85 [1.26; 2.72]	
Age (years)	43.59 (14.13)		38.34 (14.22)		0.98 [0.96; 0.99]	**<0.001**	0.98 [0.96; 0.99]	**<0.001**	0.98 [0.96; 0.99]	**<0.001**
Education level						0.99		0.52		0.43
<12 years of education	43	20.9	73	20.9						
≥12 years of education	163	79.1	276	79.1						
Professional activity						**<0.001**		**<0.001**		0.11
No	76	31.8	197	48.4	1		1			
Yes	163	68.2	210	51.6	0.50 [0.36; 0.69]		0.52 [0.37; 0.73]			
Marital status						**<0.001**		**<0.001**		0.13
In a couple	166	69.5	218	53.6	1		1			
Single	73	30.5	189	46.4	1.97 [1.41; 2.76]		1.91 [1.35; 2.70]			
Children						**0.001**		**0.001**		0.29
No	58	31.2	169	45.7	1		1			
Yes	128	68.8	201	54.3	0.54 [0.37; 0.78]		0.53 [0.36; 0.78]			
Current eating disorder						0.89		0.98		0.72
No	174	92.6	356	92.2						
Yes	14	7.4	30	7.8						
Current anxious disorder						0.51		0.69		0.83
No	107	44.8	171	42.1						
Yes	132	55.2	235	57.9						
Lifetime alcohol dependence/abuse						**0.004**		**0.007**		0.10
No	199	83.3	298	73.4	1		1		1	
Yes	40	16.7	108	26.6	1.80 [1.20; 2.70]		1.77 [1.17; 2.68]		1.45 [0.93; 2.23]	
Lifetime substance dependence/abuse						0.07		0.09		0.50
No	208	87.4	330	81.9	1		1			
Yes	30	12.6	73	18.1	1.53 [0.97; 2.43]		1.51 [0.94; 2.43]			
Smoking						0.40		0.40		0.75
No	88	47.1	140	41.8						
Yes	79	42.2	162	48.4						
Ex-smoker	20	10.7	33	9.9						
Lifetime SA						**<0.001**		**<0.001**		**<0.00**1*
No	124	51.9	121	29.9	1		1		1	
Yes	115	48.1	284	70.1	2.53 [1.82; 3.52]		2.45 [1.74; 3.44]		2.50 [1.76; 3.55]	
Number of lifetime SA *N* = 617	1.07 (1.86)		2.12 (4.22)		1.20 [1.09; 1.33]	**<0.001**	1.20 [1.09; 1.33]	**<0.001**	1.21 [1.09; 1.34]	**<0.00**1*
Age at first SA (years) *N* = 362	32.08 (14.74)		29.11 (14.51)		0.99 [0.97; 1.00]	0.09		0.18		0.31*
RRRS-risk score, last SA before inclusion *N* = 184	7.00 (2.33)		7.11 (2.16)			0.78		0.72		0.34*
RRRS-rescue score, last SA before inclusion *N* = 170	12.77 (1.54)		12.25 (2.16)			0.18		0.20	0.82 [0.66; 1.02]	0.08*
RRRS score ratio, last SA before inclusion *N* = 167	35.33 (8.46)		36.75 (8.48)			0.38		0.36		0.11*
SIS-planning score, last SA before inclusion *N* = 203	4.51 (2.42)		5.57 (3.74)		1.10 [0.99; 1.23]	0.09	1.10 [0.98; 1.23]	0.10	1.10 [0.98; 1.23]	0.10*
SIS-conceptualization score, last SA before inclusion *N* = 197	6.35 (4.48)		9.16 (3.57)		1.20 [1.09; 1.32]	**<0.001**	1.19 [1.08; 1.31]	**<0.001**	1.19 [1.08; 1.31]	**<0.00**1*
SIS, total score, last SA before inclusion *N* = 185	10.68 (5.56)		14.46 (6.35)		1.11 [1.04; 1.18]	**0.002**	1.10 [1.03; 1.18]	**0.004**	1.10 [1.03; 1.18]	**0.0**04*
BDI total score *N* = 529	18.38 (7.29)		20.76 (7.01)		1.05 [1.02; 1.07]	**<0.001**		NA	1.05 [1.02; 1.08]	0.001**
BDI without SI item *N* = 529	17.74 (6.99)		19.29 (6.46)		1.04 [1.01; 1.06]	**0.009**		NA	1.04 [1.01; 1.07]	**0.01**
IDSC30 total score *N* = 457	36.20 (7.85)		40.50 (8.47)		1.07 [1.04; 1.09]	**<0.001**		NA	1.07 [1.04; 1.10]	**<0.00**1**
IDSC30 without SI item *N* = 455	35.52 (7.66)		37.89 (8.41)		1.04 [1.01; 1.06]	**0.004**		NA	1.04 [1.01; 1.07]	0.004**
QIDS total score *N* = 460	16.41 (4.69)		18.32 (4.74)		1.09 [1.04; 1.13]	**<0.001**		NA	1.09 [1.05; 1.14]	<0.001**
QIDS without SI item *N* = 460	15.52 (4.32)		16.35 (4.33)		1.04 [0.99; 1.09]	0.06		NA	1.06 [1.01; 1.11]	0.02**
Depression severity						**<0.001**		NA		**<0.001**
Moderate	154	64.4	156	38.3	1				1	
Severe	85	35.6	251	61.7	2.92 [2.09; 4.06]				2.87 [2.03; 4.06]	
STAI-A total score *N* = 171	61.38 (9.73)		61.15 (10.01)			0.89		0.33		0.76
STAI-B total score *N* = 182	61.40 (9.16)		62.86 (8.06)			0.27		0.69		0.45
VAS current psychological pain *N* = 501	5.91 (2.60)		6.36 (2.71)		1.06 [0.99; 1.14]	0.07		0.20		0.19
VAS usual psychological pain *N* = 500	7.09 (2.09)		7.68 (1.78)		1.17 [1.07; 1.29]	**0.002**	1.14 [1.03; 1.25]	**0.01**	1.16 [1.05; 1.29]	**0.005**
VAS maximum psychological pain *N* = 502	8.09 (2.13)		8.85 (1.53)		1.26 [1.14; 1.40]	**<0.001**	1.22 [1.10; 1.36]	**<0.001**	1.21 [1.08; 1.36]	**0.001**
VAS current physical pain *N* = 501	3.45 (2.86)		3.09 (3.00)			0.20		0.23		0.40
VAS usual physical pain *N* = 500	4.21 (2.98)		3.79 (3.01)			0.13		0.12		0.43
VAS maximum physical pain *N* = 500	5.01 (3.22)		4.68 (3.32)			0.29		0.21		0.54
VAS current suicidal ideation *N* = 501	1.73 (2.78)		3.61 (3.52)		1.21 [1.13; 1.29]	**<0.001**	1.19 [1.12; 1.27]	**<0.001**	1.18 [1.10; 1.26]	**<0.001**
VAS usual suicidal ideation *N* = 501	3.17 (3.14)		6.21 (2.92)		1.36 [1.27; 1.45]	**<0.001**	1.34 [1.25; 1.43]	**<0.001**	1.33 [1.24; 1.42]	**<0.001**
VAS maximum suicidal ideation *N* = 501	4.22 (3.77)		7.69 (2.98)		1.31 [1.24; 1.39]	**<0.001**	1.30 [1.23; 1.38]	**<0.001**	1.28 [1.21; 1.36]	**<0.001**
BSSI score *N* = 173	3.59 (4.98)		15.26 (9.28)		1.21 [1.14; 1.28]	**<0.001**	1.21 [1.14; 1.28]	**<0.001**	1.21 [1.14; 1.29]	**<0.001**
BHS *N* = 186	10.37 (5.33)		12.61 (5.41)		1.07 [1.02; 1.15]	**0.02**	1.07 [1.00; 1.13]	**0.05**	1.08 [1.00; 1.15]	**0.04**
RFLI total score *N* = 169	185.81 (42.84)		154.87 (44.57)		0.99 [0.98; 0.99]	**<0.001**	0.99 [0.98; 0.99]	**<0.001**	0.99 [0.98; 0.99]	**0.005**
ESUL score *N* = 214	46.75 (10.86)		51.69 (10.17)		1.05 [1.02; 1.08]	**0.003**	1.04 [1.01; 1.07]	**0.01**	1.04 [1.00; 1.07]	**0.05**
BIS total score *N* = 179	51.23 (16.59)		49.22 (13.52)			0.38		0.43		0.20
ALS total score *N* = 127	1.42 (0.61)		1.66 (0.50)		2.24 [1.11; 4.53]	**0.02**	1.88 [0.90; 3.93]	0.10		0.16
AIM total score *N* = 155	3.83 (0.68)		3.83 (0.49)			0.99		0.91		0.93
CTQ physical abuse						**0.03**		**0.04**		0.09
None/low	140	86.4	266	78.2	1		1		1	
Moderate/severe	22	13.6	74	21.8	1.77 [1.06; 2.97]		1.74 [1.03; 2.95]		1.62 [0.93; 2.80]	
CTQ physical neglect						**0.02**		**0.05**		0.12
None/low	152	93.8	290	86.8	1		1			
Moderate/severe	10	6.2	44	13.2	2.31 [1.13; 4.71]		2.10 [1.02; 4.34]			
CTQ emotional abuse						0.10		0.18		0.30
None/low	119	73.5	223	66.2						
Moderate/severe	43	26.5	114	33.8						
CTQ emotional neglect						0.81		0.91		0.81
None/low	101	62.7	207	61.6						
Moderate/severe	60	37.3	129	38.4						
CTQ sexual abuse						0.23		0.25		0.25
None/low	140	86.4	280	82.1						
Moderate/severe	22	13.6	61	17.9						
MARS total score *N* = 450	5.75 (2.23)		5.66 (2.29)			0.70		0.77		0.72
Psychotropic intake***						0.14		0.29		0.71
No	37	20.0	50	15.0						
Yes	148	80.0	284	85.0						
Anxiolytic/hypnotic intake***						**0.02**		0.06		0.21
No	50	27.0	61	18.3	1		1			
Yes	135	73.0	273	81.7	1.66 [1.08; 2.54]		1.52 [0.98; 2.36]			
Antidepressant intake***						0.88		0.87		0.92
No	73	39.5	134	40.1						
Yes	112	60.5	200	59.9						
Antiepileptic intake***						0.07		0.14		0.19
No	165	89.2	313	93.7	1					
Yes	20	10.8	21	6.3	0.55 [0.29; 1.05]					
Antipsychotic intake***						**0.02**		0.06		0.14
No	107	57.8	156	46.7	1		1			
Yes	78	42.2	178	53.3	1.57 [1.09; 2.25]		1.43 [0.99; 2.08]			
Mood stabilizer intake***						**0.03**		0.08		0.16
No	99	53.5	145	43.4	1		1			
Yes	86	46.5	189	56.6	1.50 [1.05; 2.15]		1.39 [0.96; 2.01]			
Antalgic intake***						0.83		0.70		0.59
No	164	88.6	294	88.0						
Yes	21	11.4	40	12.0						

*Note*: Model 0, Crude association; Model 1, Adjusted for depression severity; Model 2, Adjusted for depression severity, age, sex, and lifetime SA. *Not adjusted for lifetime SA (because these variables are only for patients with lifetime SA); **not adjusted for depression severity; ***Classification according to the CIM-10: Psychotropics: N05; Anxiolytics/hypnotics: N05B and N05C; Antidepressants: N06A; Antiepileptics: N03A; Antipsychotics: N05A; Mood stabilizers: N03A and N05A; Antalgics: N02.

Abbreviations: AIM, affective intensity measure; ALS, affective lability scale; BDI, self-rated Beck Depression Inventory; BHS, Beck hopelessness scale; BIS, Barratt impulsiveness scale; BSSI, Beck Scale for Suicide Ideation; CTQ, childhood trauma questionnaire; ESUL, University of California, Los Angeles Loneliness Scale; IDSC30, clinician-rated 30-item inventory depression symptomatology; MARS, medication adherence report scale; QIDS, self-rated quick inventory of depressive symptomatology; RFLI, reasons for living inventory; RRRS, risk/rescue rating scale; SIS, suicidal intent scale; STAI, anxiety using the state/trait anxiety inventory; VAS, visual analog scales.

Regarding follow-up, as this was a naturalistic cohort (treatment as usual according to the clinician’s judgment), patients came back for routine follow-up visits (number and frequency in function of the patient’s state). Therefore, psychometric tools (e.g., hopelessness scale) were used and other data (e.g., current treatment, change in sociodemographic data) were collected only if deemed necessary by the psychiatrists for the proper management of the patient. The occurrence (date and nature) of SA and hospitalization for SI (emergency room, psychiatric ward, or other wards) were recorded by psychiatrists during the routine follow-up visits and were also extracted from the patients’ hospital records. Using two source types reduced the risk of oversight and/or mistakes (e.g., using only medical records may miss nonhospitalized SA). In addition, depression severity was monitored at 3, 6, and 12 months with the scales used at baseline (BDI, IDSC30, and QIDS) only if the patients accepted to fill them in. Thus, the patient sample may vary during the follow-up; however, as this was a naturalistic study, all patients who came back for routine care were included in the follow-up analyses. The numbers of patients who accepted to complete depression scales during their routine follow-up visits are reported in Table 2.

**Table 2.** Remission of depression during the 1-year follow-up.

Variables	Suicidal depression				
	No		Yes		*p-*value
	*N* (%)		*N* (%)		
At 3 months	94		142		
BDI					0.34
≤9	27	33.8	38	40.9	
>9	53	66.3	55	59.1	
IDSC30					0.46
≤13	27	50.9	51	44.7	
>13	26	49.1	63	55.3	
QIDS					0.81
≤5	8	20	21	21.9	
>5	32	80	75	78.1	
At 6 months	87		151		
BDI					0.41
≤9	22	37.3	32	44.4	
>9	37	62.7	40	55.6	
IDSC30					0.51
≤13	27	54	72	59.5	
>13	23	46	49	40.5	
QIDS					0.20
≤5	6	21.4	26	34.7	
>5	22	78.6	49	65.3	
At 12 months	56		67		
BDI					0.79
≤9	23	46.9	23	44.2	
>9	26	53.1	29	55.8	
IDSC30					0.75
≤13	14	53.8	26	50	
>13	12	46.2	26	50	
QIDS					0.84
≤5	6	30	11	27.5	
>5	14	70	29	72.5	

Abbreviations: BDI, Beck Depression Inventory; IDSC30, clinician-rated 30-item inventory depression symptomatology; QIDS, quick inventory of depressive symptomatology.

**Figure 1.**
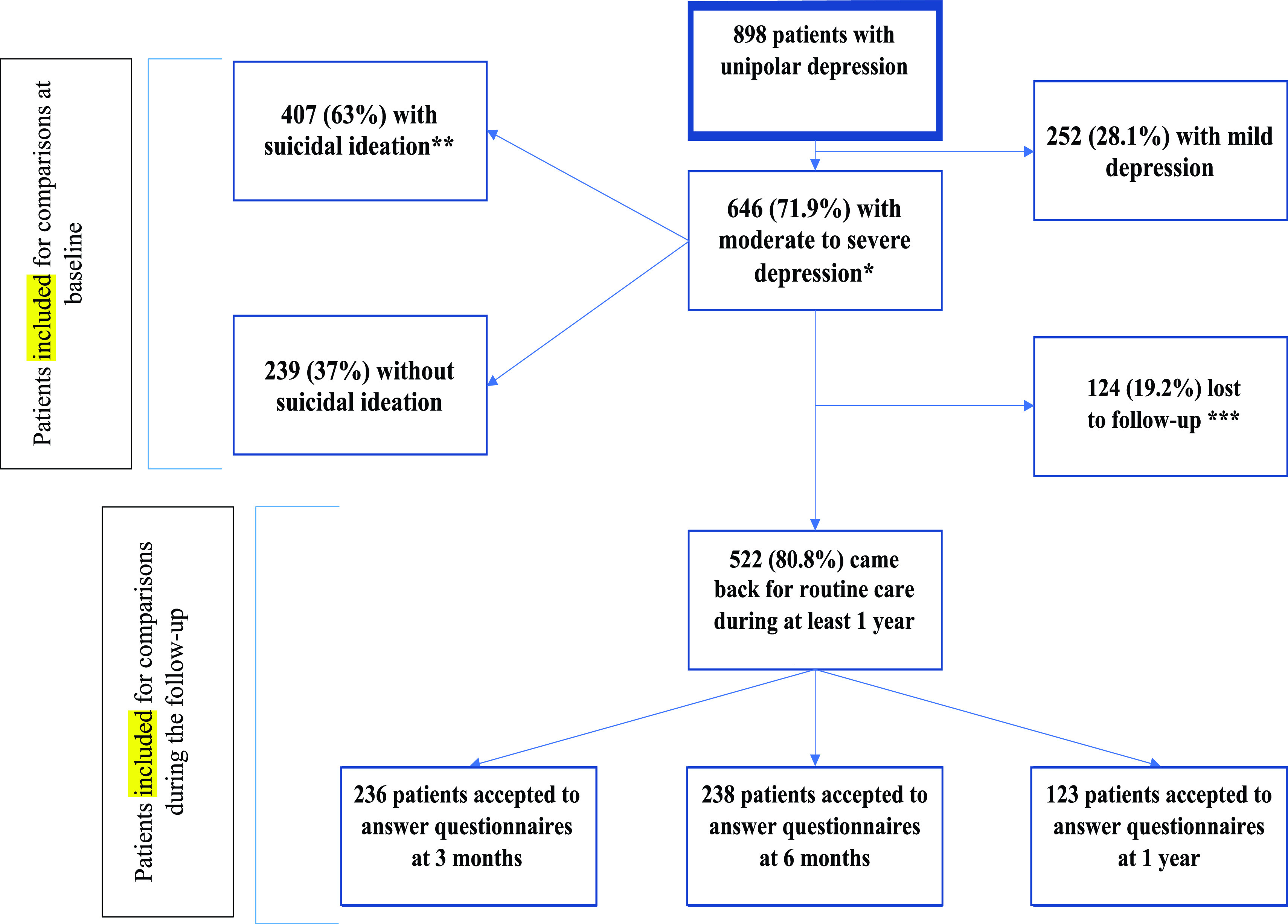
Flowchart of patients selection for analysis. *Defined by an IDSC30 score ≥ 24, or a QIDS score ≥ 11, or a BDI score ≥ 19. **Defined by a score ≥2 for the suicide item of the IDSC30, the QIDS, or the BDI. ***Never came back even for routine care.

Patients who did not come back for follow-up visits and without SA and/or hospitalization for SI (record in their medical records) during the 1-year follow-up period were considered as lost to follow-up (*N* = 124, 19.2% of patients with moderate/severe depression) and were not included in follow-up analyses (see flowchart, Figure 1). No significant difference in SI and depression severity was observed between patients lost or not to follow-up.

### Definition of suicidal depression

To match the inclusion criteria of clinical trials on anti-suicidal agents [49,50], moderate to severe depression was defined by an IDSC30 score ≥ 24, or a QIDS score ≥ 11, if the IDSC30 score was not available, or a BDI score ≥ 19, if both IDSC30 and QIDS scores were missing. These scores correspond to the usual cut-off values used to define moderate depression with these scales [51,52].

SI presence was defined using a single suicide item from a depression rating scale (clinician-rated or self-reported). A single suicide item from a depression rating scale, either clinician-rated or self-reported, is a valid approach to assess SI, compared with the Beck Scale for SI, and has been used in previous clinical studies [53–56]. Thus, SI presence was defined by a score ≥ 2 for the suicide item of the IDSC30 (IDSC30-SI), the QIDS (QIDS-SI), or the BDI (BDI-SI). This BDI-SI cut-off was previously used [23] and is associated with a 7-fold higher risk of future death by suicide [57].

Therefore, suicidal depression was defined as moderate to severe depression with SI. Nonsuicidal depression was defined as moderate to severe depression without SI.

### Definition of depression severity

As three different depression scales were used, to adjust the statistical models on depression severity, this variable was categorized into two categories: moderate depression, and severe depression. Severe depression was defined by an IDSC30 score ≥ 37, a QIDS score ≥ 16, and a BDI score ≥ 30, according to clinical practice.

### Definition of depression remission

Depression remission during the follow-up was defined by an IDSC30 score ≤ 13, a QIDS score ≤ 5, and a BDI score ≤ 9, according to clinical practice.

### Statistical analyses

Categorical variables were presented as percentages and quantitative variables as means with standard deviation (SD).

Baseline data between patients’ groups were compared with a univariate binary logistic regression model (model 0) and the likelihood-ratio test (LRT), and crude odds ratios (OR) and 95% confidence intervals (CI) were estimated. The outcome was “suicidal depression” and its association with each variable was tested individually. Then, multivariate binary logistic regression models and LRT were performed, and adjusted OR and 95% CI were estimated. The outcome was “suicidal depression” and its association with each variable, independently of potential confounders included in the models, was tested individually. As some scales were not filled in by the whole sample, potential confounders were chosen according to their association with the outcome in the univariate model and to their clinical interest. The first multivariate model (model 1) was adjusted for depression severity (i.e., moderate or severe) to test whether associations were explained by the depression severity or presence of SI, because SI and depression severity were linked. This method was also used in previous studies [23,58]. A second multivariate model (model 2) was adjusted for depression severity, age, sex, and lifetime SA, because these variables were associated with SI. A third multivariate model (model 3, Supplementary Material) was adjusted for depression severity, age, sex, lifetime SA and maximum psychological pain because maximum psychological pain has been associated with SI [59].

Remission of depression during the follow-up was compared between groups with a univariate binary logistic regression model and LRT. Patients included in this analysis were those who filled in questionnaires during the routine care follow-up visits: 236 patients at 3 months, 238 patients at 6 months, and 123 patients at 1 year.

The risk of actual SA and of suicidal events (SE) (i.e., actual SA, aborted SA, interrupted SA, hospitalization for SI) during the follow-up was compared between groups using a univariate binary logistic regression model (model 0) and LRT, followed by a multivariate binary logistic regression model (model 1) and LRT adjusted for baseline depression severity, age, and sex. Two additional multivariate binary logistic regression models and LRT were run: (a) one adjusted for age, sex, baseline maximum psychological pain, and baseline depression severity (model 2), and (b) one adjusted for age, sex, lifetime SA, marital status, professional activity, baseline maximum psychological pain, and baseline depression severity (model 3).

Univariate and multivariate Cox regression models were used to compare the time to actual SA or SE in patients with suicidal and nonsuicidal depression. The outcome was the occurrence of an actual SA or a SE (one Cox model for each outcome). The chance of survival was compared in patients with suicidal and nonsuicidal depression. One Cox regression model was adjusted for baseline depression severity, age, sex, and lifetime SA. The other Cox regression model was adjusted for baseline depression severity, age, sex, lifetime SA, and maximum baseline psychological pain. Hazard ratios (HR) and 95% CI were estimated; survival curves were generated. As mentioned above, patients that never came back for routine care and without SA recorded in their hospital record during the follow-up were excluded from this analysis.

As patients with history of SA are at higher risk of future SA and SI, all our analyses were adjusted for lifetime SA to exclude its effect on the association between SI and the tested outcomes. The risk of SA during the follow-up was assessed in patients with and without history of lifetime SA.

Missing data were not imputed. The significance level was set at *p* < 0.05. Analyses were performed with the SPSS statistical software (version 26.0).

## Results

Among the 898 inpatients with depression, 646 patients (71.9%) had moderate to severe depression. The mean (SD) age of these 646 patients was 40.28 (14.40) years, and 31.8% were men. The mean (SD) IDSC30, QIDS and BDI scores were 39.05 (8.51), 17.71 (4.80), and 19.82 (7.21), respectively. Moreover, at inclusion, 407 patients (63% of 646) had current SI (suicidal depression) and 239 patients (37%) did not (nonsuicidal depression), and 399 patients (61.8%) had lifetime history of SA. Data on SE during the 1-year follow-up were available for 522 (80.8%) patients.

### Comparison of baseline characteristics in patients with suicidal depression and patients with nonsuicidal depression (Supplementary Table S1)

Patients with suicidal depression were younger and more likely to be men, single, with no professional activity, and without children compared with patients with nonsuicidal depression. Lifetime alcohol dependence and/or abuse were more frequent in the suicidal depression than nonsuicidal depression group.

The mean (SD) scores of the BSSI and VAS-SI (not used to define SI) were higher in patients with suicidal depression than in patients with nonsuicidal depression: BSSI = 3.59 (4.98) versus 15.26 (9.28), *p-*value < 0.001, and VAS-SI = 1.73 (2.78) versus 3.61 (3.52), *p-*value < 0.001. Similarly, lifetime SA (OR = 2.50, 95% CI = [1.76; 3.55], *p-*value < 0.001, model 2) and lifetime number of SA (1.07 (1.86) versus 2.12 (4.22), OR = 1.21, 95% CI = [1.09; 1.34], *p-*value < 0.001, model 2) were higher in patients with suicidal depression than in patients with nonsuicidal depression. Among patients with history of lifetime SA (before inclusion), patients with suicidal depression reported higher suicidal intent (SIS total score: OR = 1.10, 95% CI = [1.03; 1.18], *p-*value = 0.004, model 2) during the last SA than nonsuicidal patients.

Levels of depression, usual and maximum psychological pain (maximum VAS-psychological pain OR = 1.21, 95% CI = [1.08; 1.36], *p-*value = 0.01, model 2), hopelessness (OR = 1.08, 95% CI = [1.00; 1.15], *p-*value = 0.04, model 2), feelings of loneliness (OR = 1.04, 95% CI = [1.00; 1.07], *p-*value = 0.05, model 2), and emotional lability were higher in patients with suicidal depression that with nonsuicidal depression. Similarly, patients with suicidal depression reported more often history of physical abuse and neglect (CTQ scores), and fewer reasons for living (OR = 0.99 [0.98; 0.99], *p-*value = 0.005, model 2).

Patients with suicidal depression were treated more often with anxiolytics/hypnotics, antipsychotics, and mood stabilizers than patients with nonsuicidal depression.

In the second multivariate model (model 2, adjusted for depression severity, lifetime SA, age, and sex), number of lifetime SA, suicidal intent, psychological pain, hopelessness, loneliness, and reasons for living remained significantly different between groups. In the final multivariate model (model 3, Supplementary Table S1) that included also maximum psychological pain as a potential cofounder, results did not change.

### Remission of depression in patients with suicidal and nonsuicidal depression during the 1-year follow-up (Supplementary Table S2)

The rates of depression remission during the follow-up were not different between groups. Of note, they varied in function of the used depression scale. Indeed, the rate was higher with the clinician-rated than with the self-reported scale at each follow-up visit.

### Risk of actual SA and SE (i.e., actual SA, aborted SA, interrupted SA, and hospitalization for SI) during the 1-year follow-up in patients with suicidal and nonsuicidal depression (Tables 3–5, Figures 2 and 3, and Supplementary Tables S2–S4)

**Figure 2.**
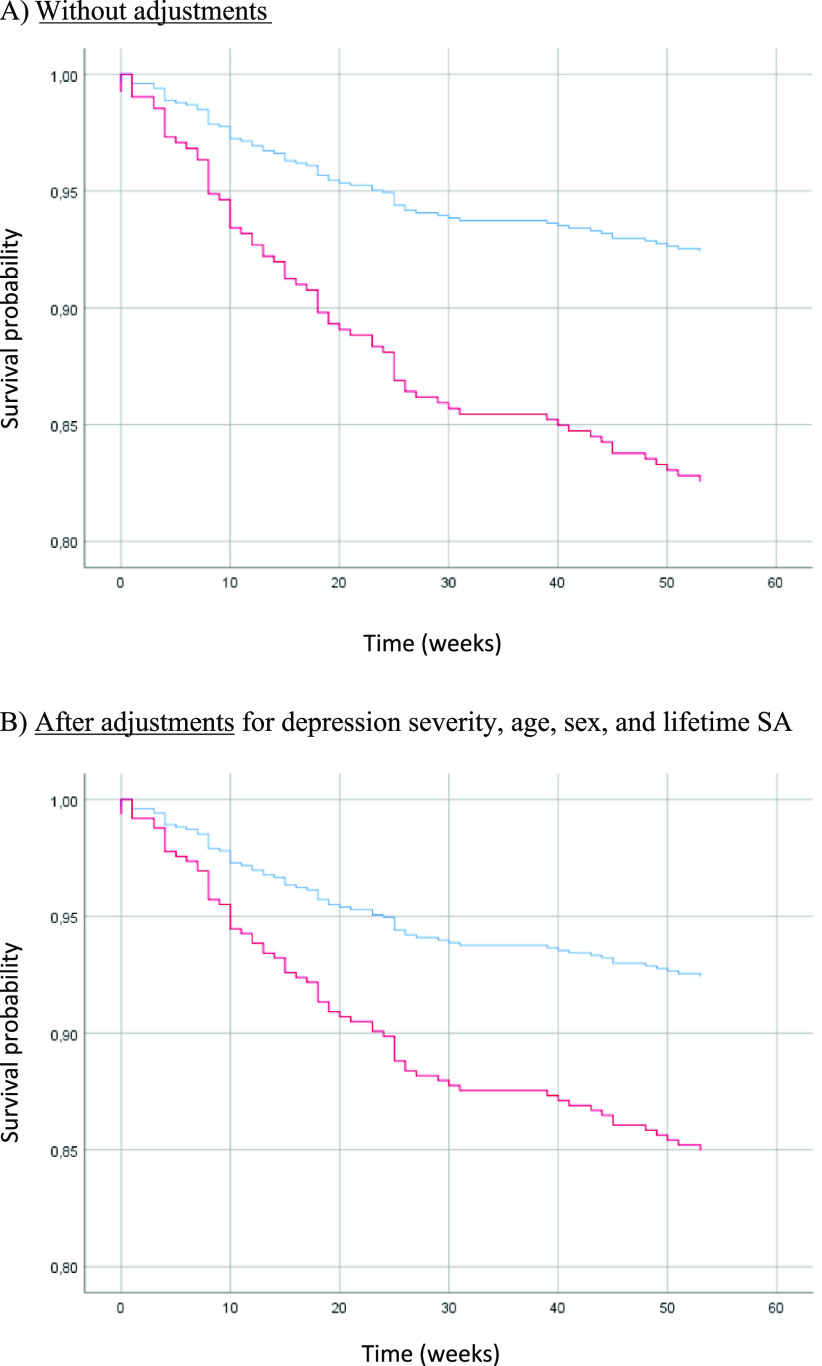
Risk of actual SA during the 1-year follow-up in patients with suicidal depression (red) and nonsuicidal depression (blue) at baseline.

**Figure 3.**
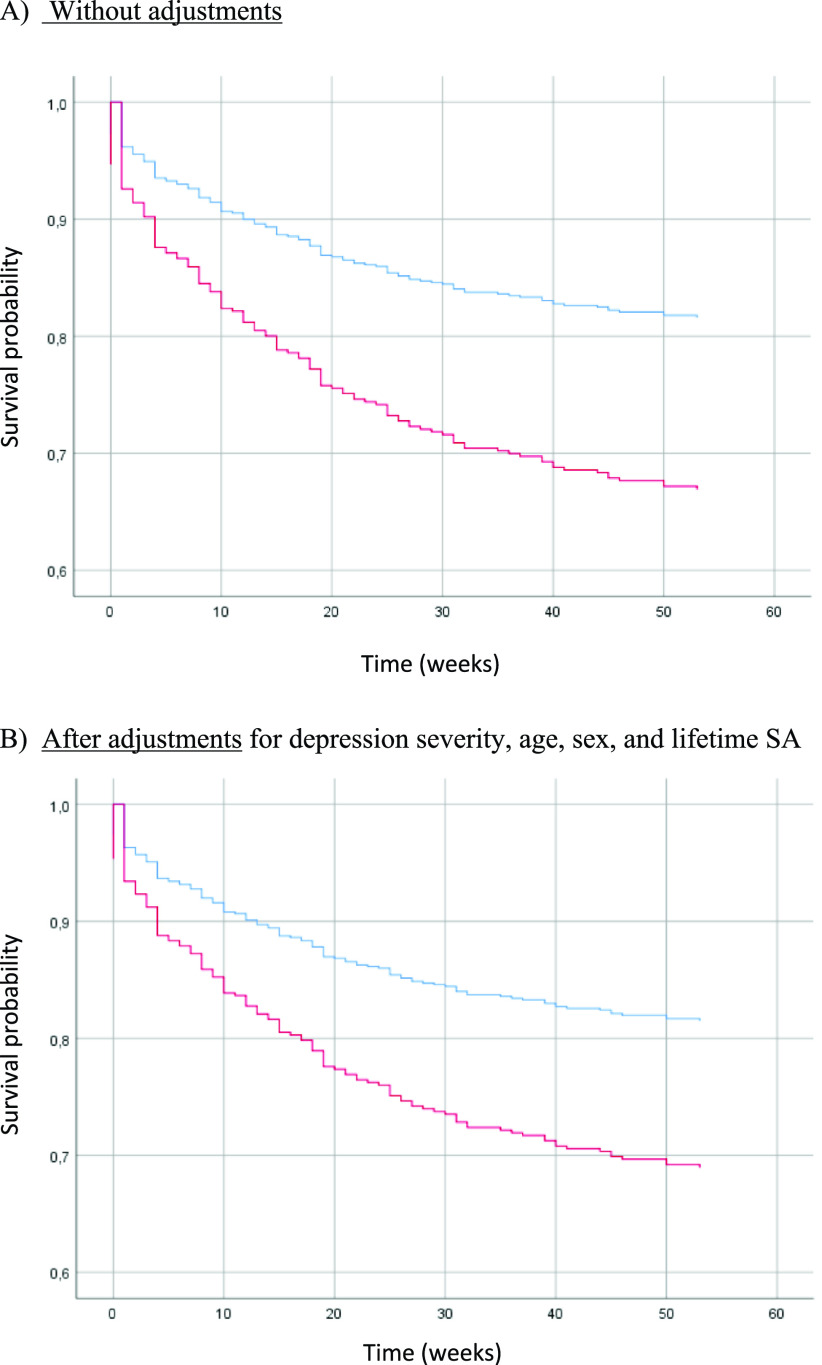
Risk of SE (actual SA, aborted SA, interrupted SA, and hospitalization for SI) during the 1-year follow-up in patients with suicidal depression (red) and nonsuicidal depression (blue) at baseline.

The 156 SE identified during the follow-up included 72 (46.2%) actual SA, 18 (11.5%) aborted or interrupted SA, and 66 (42.3%) hospitalizations for SI. Patients with suicidal depression at baseline were at higher risk of actual SA and SE during the 1-year follow-up than nonsuicidal patients in the unadjusted and adjusted models (i.e., age, sex, baseline depression severity) (model 1: OR = 2.35, 95% CI = [1.24; 4.44], *p-*value = 0.009 and OR = 1.98, 95% CI = [1.27; 3.08], *p-*value = 0.002, respectively) (Table 3). Results were similar when considering only patients with history of lifetime SA (*n* = 357). Conversely, no difference between groups was observed when only patients without history of lifetime SA (*n* = 201) were considered.

**Table 3.** Risk of actual SA and SE (i.e., actual SA, aborted SA, interrupted SA, and hospitalization for SI) during the 1-year follow-up in patients with suicidal and nonsuicidal depression.

Variables	Suicidal depression				Model 0		Model 1	
	No *N* (%)		Yes *N* (%)		OR [95% CI]	*p-*value	OR [95% CI]	*p-*value
Actual SA						**0.003**		**0.009**
No	171	92.4	275	82.6	1		1	
Yes	14	7.6	58	17.4	2.58 [1.39; 4.76]		2.35 [1.24; 4.44]	
Suicidal event*						**0.001**		**0.002**
No	147	79.5	219	65	1		1	
Yes	38	20.5	118	35	2.08 [1.37; 3.18]		1.98 [1.27; 3.08]	
New actual SA (for patients with previous SA, *N* = 357)						**0.003**		**0.006**
No	86	93.5	183	79.2	1		1	
Yes	6	6.5	48	20.8	3.76 [1.55; 9.12]		3.59 [1.45; 8.92]	
New SE (for patients with previous SA, *N* = 357)						**0.004**		**0.005**
No	73	79.3	146	62.7	1		1	
Yes	19	20.7	87	37.3	2.29 [1.29; 4.05]		2.35 [1.29; 4.27]	
First actual SA (for patients without previous SA, *N* = 201)						0.74		0.88
No	85	91.4	90	90				
Yes	8	8.6	10	10				

*Note:* Model 0, crude association; Model 1, adjusted for age, sex, and baseline depression severity. *Suicidal event: actual SA, aborted SA, interrupted SA, and hospitalization for SI.

Abbreviations: SA, suicide attempt; SE, suicide event; SI, suicidal ideation.

Due to the small sample size, in the final model (adjusted for age, sex, lifetime SA, marital status, professional activity, baseline maximum psychological pain, and baseline depression severity) the whole cohort was considered (Supplementary Table S2). In this model, patients with baseline suicidal depression were at higher risk of actual SA and SE during the follow-up (model 3: OR = 2.14, 95% CI = [1.06; 4.33], *p-*value = 0.03 and OR = 1.73, 95% CI = [1.07; 2.79], *p-*value = 0.03, respectively).

Figures 2 and 3 show the survival curves for patients with and without suicidal depression during the 1-year follow-up. This survival analysis showed that the majority of actual SA and SE occurred in the 30 weeks following inclusion. For both outcomes, baseline suicidal depression was associated with significantly poorer survival probability in the unadjusted and adjusted Cox regressions models (curve decreasing faster for the suicidal depression group). In the adjusted model, suicidal depression (at inclusion) was associated with a 2-fold higher risk of actual SA (HR = 2.07, 95% CI = (1.13; 3.77), *p-*value = 0.02) (Table 4) and a 1.8-fold higher risk of SE (HR = 1.82, 95% CI = (1.21; 2.73), *p-*value = 0.004) (Table 5) during the 1-year follow-up. Younger age also was associated with the risk of actual SA and SE during the follow-up. Results did not change after inclusion of maximum psychological pain at baseline (Supplementary Table S4).

**Table 4.** Cox regression model to estimate the risk of actual SA during the 1-year follow-up in patients with suicidal and nonsuicidal depression at baseline.

	HR (95% CI)	*p-*value
Unadjusted model		
Suicidal depression		**0.001**
No	1	
Yes	2.43 (1.36; 4.36)	
Adjusted model		
Suicidal depression		**0.02**
No	1	
Yes	2.07 (1.13; 3.77)	
Age	0.97 (0.95; 0.99)	**0.001**
Sex		0.61
Men	1	
Women	0.88 (0.52; 1.46)	
Lifetime SA		0.09
No	1	
Yes	1.59 (0.93; 2.72)	
Baseline depression severity		0.77
Moderate	1	
Severe	1.08 (0.66; 1.77)	

Abbreviation: SA, suicide attempt.

**Table 5.** Cox regression model to estimate the risk of SE (i.e., actual SA, aborted SA, interrupted SA, and hospitalization for SI) during the 1-year follow-up in patients with suicidal and nonsuicidal depression at baseline.

	HR (95% CI)	*p-*value
Unadjusted model		
Suicidal depression		**0.001**
No	1	
Yes	1.98 (1.34; 2.92)	
Adjusted model		
Suicidal depression		**0.004**
No	1	
Yes	1.82 (1.21; 2.73)	
Age	0.98 (0.96; 0.99)	**< 0.001**
Sex		0.88
Men	1	
Women	0.97 (0.68; 1.39)	
Lifetime SA		0.27
No	1	
Yes	1.23 (0.85; 1.77)	
Baseline depression severity		0.41
Moderate	1	
Severe	0.87 (0.61; 1.22)	

Abbreviations: SA, suicide attempt; SE, suicide event; SI, suicidal ideation.

## Discussion

To our knowledge, this is the first study to characterize suicidal depression (i.e., moderate to severe depression with active SI) in a large observational longitudinal cohort of inpatients. Our results indicate that patients with suicidal depression are different from nonsuicidal patients and present more severe clinical features (depression level, history of SA, psychological pain, hopelessness, feelings of loneliness, and reasons for living). In patients with history of lifetime SA before inclusion, the number of lifetime SA and suicidal intent was higher in patients with than without baseline SI. As previously done [23, 60–62], our results were adjusted for depression severity and also for history of SA to avoid their confounding effects. Therefore, the observed differences should be mainly due to this specific phenotype.

Interestingly, clinical characteristics strongly associated with suicidal depression are also strongly associated with higher suicidal risk [63]. Moreover, it has been proposed that these clinical characteristics are involved in the three-step theory (3ST) of suicide [64]. This theory integrates the “ideation-to-action-framework” according to which SI genesis and SI to SA transition are distinct phenomena [57,65]. In the 3ST, psychological pain and hopelessness (higher in patients with suicidal depression in our study) are the main clinical characteristics at the origin of SI. If psychological pain is higher than connectedness (represented by fewer reasons for living and higher levels of loneliness in our study), SI becomes stronger. Finally, if patients have the capacity to attempt suicide (e.g., access to lethal means and lifetime history of SA), they might act. This last step could be reflected by higher history of SA and number of SA in patients with suicidal depression. Thus, our results indirectly strengthen the 3ST and suggest that inpatients with active SI might be at higher suicidal risk. Therefore, targeting these specific dimensions (e.g., psychological pain and feelings of loneliness) could reduce suicide risk.

This higher suicidal risk was confirmed by our prospective analyses. During the follow-up, the risk of actual SA and SE was 2-fold and 1.8-fold higher, respectively, in patients with baseline suicidal depression. In patients with history of SA, baseline suicidal depression was associated with a 3.6-fold higher risk of actual SA and a 2.4-fold higher risk of SE. When considering only patients without history of SA, baseline suicidal depression was not associated with the risk of actual SA during the follow-up. This may be due to the limited statistical power because only 18 patients (*n* = 8 with nonsuicidal depression and *n* = 10 with suicidal depression at baseline) without lifetime history of SA attempted suicide during the follow-up. Conversely, in the final model that included the whole sample and lifetime history of SA as covariate, baseline suicidal depression was significantly associated with the risk of actual SA and SE during the follow-up. This suggests that this association is partially independent of lifetime history of SA. Furthermore, in the Cox models, lifetime history of SA was not associated with the risk of actual SA or SE, unlike baseline suicidal depression. Finally, being younger also was associated with increased risk of actual SA and SE during the follow-up, possibly because patients with suicidal depression were younger at baseline. A recent World Health Organization fact-sheet states that “a prior SA is the single most important risk factor” for suicide [66]. However, our study suggests that patients with current suicidal depression are at high risk of actual SA and SE, and this is partially independent of their history of SA. This is reinforced by recent meta-analyses showing that SI and SA are similarly associated with death by suicide [6,67]. Thus, clinicians should very carefully assess the existence of both past SA and current SI.

We also found that the depression remission rate during the follow-up was not different in patients with suicidal and nonsuicidal depression. However, at each follow-up visit, remission rates based on the clinician-rated score were higher than those based on the self-rated scores. This highlights that in suicidal patients self-rated and clinician-rated evaluations are different [68]. Clinicians may under-evaluate depression severity compared with patients [68,69]. Finally, patients with baseline suicidal depression were at greater risk of actual SA despite the optimized treatment (i.e., care in a hospital department specialized in suicidal crisis management), which led to the improvement of the depressive symptomatology, and their referral to a specialized hospital department. We could hypothesize that SI and SA are related to, but partly independent of depression and deserve a specific management. Modern psychiatry must use a new medical model in suicide. Depression itself is not a useful tool to understand the complexity of suicide, especially because some suicidal patients could be ambivalent (i.e., contemplating suicide but still attached to life) [70].

One of the main limitations of this study is the high number of missing data. However, our naturalistic study reflects daily clinical practice. Thus, by including suicidal patients our results reflect more “the real world” than the findings obtained in randomized controlled trials. Moreover, as we found significant associations despite the missing data, we may have underestimated these associations. Another limitation is the lack of data (e.g., SA method, therapeutic failure) on the SA or SE that occurred during follow-up. Moreover, the naturalistic design of this study did not allow measuring variables, such as depression, psychological pain, regularly during the follow-up period. Finally, the presence of personality disorders (e.g., borderline personality disorder) was not assessed. However, it is unlikely that many patients with baseline suicidal depression had a borderline personality disorder.

In conclusion, our results suggest that suicidal depression could be a specific phenotype of depression with more severe clinical characteristics and higher risk of SA or SE, despite optimal care (i.e., care in a hospital department specialized in suicidal crisis management). Our results also contribute to the hypothesis that depression and SI are related but can also have independent physiopathology. Finally, clinicians should monitor closely patients with suicidal depression, whatever their history of SA and SI.

## Data Availability

The data that support the findings of this study are available on request from the corresponding author, B.N. The data are not publicly available due to restrictions (the containing information that could compromise the privacy of research participants).

## Supplementary Materials

To view supplementary material for this article, please visit http://doi.org/10.1192/j.eurpsy.2022.16. click here to view supplementary material
