# Supplementary material for: Characterization of suicidal depression: A 1 year prospective study
Source: Eur Psychiatry. 2022 Apr 18;65(1):e24. doi: 10.1192/j.eurpsy.2022.16 (PMC9058444; doi:10.1192/j.eurpsy.2022.16)
Supplement: Supplementary file 1 [file S0924933822000165sup001.docx]

Suppl Table 1: Baseline characteristics of patients with suicidal depression and patients with non-suicidal depression

|  | Suicidal depression | | | | | | | Model 3 | |
| --- | --- | --- | --- | --- | --- | --- | --- | --- | --- |
| Variables | No  N = 239  Mean (sd)/N (%) | | | | Yes  N = 407  Mean (sd)/N (%) | | | OR [95% CI] | *p-value* |
| Sex |  | | | |  | | |  | **0.001** |
| Women | 176 | | 73.6 | | 268 | | 65.8 | 1 |  |
| Men | 63 | | 26.4 | | 139 | | 34.2 | 2.18 [1.40; 3.40] |  |
| Age (years) | 43.59 (14.13) | | | | 38.34 (14.22) | | | 0.99 [0.97; 1.00] | **0.05** |
| Education level |  | |  | |  |  | |  | 0.35 |
| <12 years of education | 43 | | 20.9 | | 73 | 20.9 | |  |  |
| ≥12 years of education | 163 | | 79.1 | | 276 | 79.1 | |  |  |
| Professional activity |  | |  | |  |  | |  | **0.007** |
| No | 76 | | 31.8 | | 197 | 48.4 | | 1 |  |
| Yes | 163 | | 68.2 | | 210 | 51.6 | | 0.53 [0.34; 0.84] |  |
| Marital status |  | |  | |  |  | |  | **0.007** |
| In a couple | 166 | | 69.5 | | 218 | 53.6 | | 1 |  |
| Single | 73 | | 30.5 | | 189 | 46.4 | | 1.90 [1.19; 3.02] |  |
| Children |  | |  | |  |  | |  | 0.15 |
| No | 58 | | 31.2 | | 169 | 45.7 | |  |  |
| Yes | 128 | | 68.8 | | 201 | 54.3 | |  |  |
| Current eating disorder |  | |  | |  |  | |  | 0.37 |
| No | 174 | | 92.6 | | 356 | 92.2 | |  |  |
| Yes | 14 | | 7.4 | | 30 | 7.8 | |  |  |
| Current anxious disorder |  | |  | |  |  | |  | 0.44 |
| No | 107 | | 44.8 | | 171 | 42.1 | |  |  |
| Yes | 132 | | 55.2 | | 235 | 57.9 | |  |  |
| Lifetime alcohol dependence/abuse |  | |  | |  |  | |  | 0.12 |
| No | 199 | | 83.3 | | 298 | 73.4 | |  |  |
| Yes | 40 | | 16.7 | | 108 | 26.6 | |  |  |
| Lifetime substance dependence/abuse |  | |  | |  |  | |  | 0.73 |
| No | 208 | | 87.4 | | 330 | 81.9 | |  |  |
| Yes | 30 | | 12.6 | | 73 | 18.1 | |  |  |
| Smoking |  | |  | |  |  | |  | 0.68 |
| No | 88 | | 47.1 | | 140 | 41.8 | |  |  |
| Yes | 79 | | 42.2 | | 162 | 48.4 | |  |  |
| Ex-smoker | 20 | | 10.7 | | 33 | 9.9 | |  |  |
| Lifetime SA |  | |  | |  |  | |  | **<0.001*** |
| No | 124 | | 51.9 | | 121 | 29.9 | | 1 |  |
| Yes | 115 | | 48.1 | | 284 | 70.1 | | 2.31 [1.55; 3.46] |  |
| Number of lifetime SA  N = 617 | 1.07 (1.86) | | | | 2.12 (4.22) | | | 1.21 [1.06; 1.37] | **0.004*** |
| Age at first SA (years)  N = 362 | 32.08 (14.74) | | | | 29.11 (14.51) | | |  | 0.49* |
| RRRS-risk score, last SA before inclusion  N = 184 | 7.00 (2.33) | | | | 7.11 (2.16) | | |  | 0.77* |
| RRRS-rescue score, last SA before inclusion  N = 170 | 12.77 (1.54) | | | | 12.25 (2.16) | | |  | 0.12* |
| RRRS score ratio,  last SA before inclusion  N = 167 | 35.33 (8.46) | | | | 36.75 (8.48) | | |  | 0.27* |
| SIS-planning score, last SA before inclusion  N = 203 | 4.51 (2.42) | | | | 5.57 (3.74) | | |  | 0.11* |
| SIS-conceptualization score, last SA before inclusion  N = 197 | 6.35 (4.48) | | | | 9.16 (3.57) | | | 1.17 [1.06; 1.30] | **0.002*** |
| SIS, total score  last SA before inclusion  N = 185 | 10.68 (5.56) | | | | 14.46 (6.35) | | | 1.09 [1.02; 1.17] | **0.02*** |
| BDI total score  N = 529 | 18.38 (7.29) | | | | 20.76 (7.01) | | | 1.05 [1.02; 1.09] | **0.002**** |
| BDI without SI item  N = 529 | 17.74 (6.99) | | | | 19.29 (6.46) | | | 1.05 [1.01; 1.08] | **0.01**** |
| IDSC30 total score  N = 457 | 36.20 (7.85) | | | | 40.50 (8.47) | | | 1.07 [1.04; 1.10] | **<0.001**** |
| IDSC30 without SI item  N = 455 | 35.52 (7.66) | | | | 37.89 (8.41) | | | 1.04 [1.01; 1.07] | **0.006**** |
| QIDS total score  N = 460 | 16.41 (4.69) | | | | 18.32 (4.74) | | | 1.07 [1.02; 1.12] | **0.006**** |
| QIDS without SI item  N = 460 | 15.52 (4.32) | | | | 16.35 (4.33) | | |  | 0.29****** |
| Depression severity |  | | | |  | | |  | **<0.001** |
| Moderate | 154 | 64.4 | | 156 | | 38.3 | | 1 |  |
| Severe | 85 | 35.6 | | 251 | | 61.7 | | 2.18 [1.55; 3.46] |  |
| STAI-A total score  N = 171 | 61.38 (9.73) | | | | 61.15 (10.01) | | |  | 0.51 |
| STAI-B total score  N = 182 | 61.40 (9.16) | | | | 62.86 (8.06) | | |  | 0.83 |
| VAS current psychological pain  N = 501 | 5.91 (2.60) | | | | 6.36 (2.71) | | |  | 0.19*** |
| VAS usual psychological pain  N = 500 | 7.09 (2.09) | | | | 7.68 (1.78) | | | 1.16 [1.05; 1.29] | **0.005***** |
| VAS maximum psychological pain  N = 502 | 8.09 (2.13) | | | | 8.85 (1.53) | | | 1.21 [1.08; 1.36] | **0.001** |
| VAS current physical pain  N = 501 | 3.45 (2.86) | | | | 3.09 (3.00) | | |  | 0.26 |
| VAS usual physical pain  N = 500 | 4.21 (2.98) | | | | 3.79 (3.01) | | |  | 0.20 |
| VAS maximum physical pain  N = 500 | 5.01 (3.22) | | | | 4.68 (3.32) | | |  | 0.21 |
| VAS current suicidal ideation  N = 501 | 1.73 (2.78) | | | | 3.61 (3.52) | | | 1.16 [1.09; 1.24] | **<0.001** |
| VAS usual suicidal ideation  N = 501 | 3.17 (3.14) | | | | 6.21 (2.92) | | | 1.32 [1.22; 1.41] | **<0.001** |
| VAS maximum suicidal ideation  N = 501 | 4.22 (3.77) | | | | 7.69 (2.98) | | | 1.28 [1.20; 1.37] | **<0.001** |
| BSSI score  N = 173 | 3.59 (4.98) | | | | 15.26 (9.28) | | | 1.22 [1.14; 1.30] | **<0.001** |
| BHS  N = 186 | 10.37 (5.33) | | | | 12.61 (5.41) | | |  | 0.15 |
| RFLI total score  N = 169 | 185.81 (42.84) | | | | 154.87 (44.57) | | | 0.99 [0.98; 0.99] | **0.02** |
| ESUL score  N = 214 | 46.75 (10.86) | | | | 51.69 (10.17) | | |  | 0.06 |
| BIS total score  N = 179 | 51.23 (16.59) | | | | 49.22 (13.52) | | |  | 0.28 |
| ALS total score  N = 127 | 1.42 (0.61) | | | | 1.66 (0.50) | | |  | 0.12 |
| AIM total score  N = 155 | 3.83 (0.68) | | | | 3.83 (0.49) | | |  | 0.60 |
| CTQ Physical abuse |  |  | | |  |  | |  | 0.16 |
| None/low | 140 | 86.4 | | | 266 | 78.2 | |  |  |
| Moderate/severe | 22 | 13.6 | | | 74 | 21.8 | |  |  |
| CTQ Physical neglect |  |  | | |  |  | |  | 0.10 |
| None/low | 152 | 93.8 | | | 290 | 86.8 | |  |  |
| Moderate/severe | 10 | 6.2 | | | 44 | 13.2 | |  |  |
| CTQ Emotional abuse |  |  | | |  |  | |  | 0.41 |
| None/low | 119 | 73.5 | | | 223 | 66.2 | |  |  |
| Moderate/severe | 43 | 26.5 | | | 114 | 33.8 | |  |  |
| CTQ Emotional neglect |  |  | | |  |  | |  | 0.75 |
| None/low | 101 | 62.7 | | | 207 | 61.6 | |  |  |
| Moderate/severe | 60 | 37.3 | | | 129 | 38.4 | |  |  |
| CTQ Sexual abuse |  |  | | |  |  | |  | 0.35 |
| None/low | 140 | 86.4 | | | 280 | 82.1 | |  |  |
| Moderate/severe | 22 | 13.6 | | | 61 | 17.9 | |  |  |
| MARS total score  N = 450 | 5.75 (2.23) | | | | 5.66 (2.29) | | |  | 0.70 |
| Psychotropic intake**** |  | |  | |  | |  |  | 0.79 |
| No | 37 | | 20.0 | | 50 | | 15.0 |  |  |
| Yes | 148 | | 80.0 | | 284 | | 85.0 |  |  |
| Anxiolytic/hypnotic intake**** |  | |  | |  | |  |  | 0.23 |
| No | 50 | | 27.0 | | 61 | | 18.3 |  |  |
| Yes | 135 | | 73.0 | | 273 | | 81.7 |  |  |
| Antidepressant intake**** |  | |  | |  | |  |  | 0.78 |
| No | 73 | | 39.5 | | 134 | | 40.1 |  |  |
| Yes | 112 | | 60.5 | | 200 | | 59.9 |  |  |
| Antiepileptic intake**** |  | |  | |  | |  |  | 0.19 |
| No | 165 | | 89.2 | | 313 | | 93.7 |  |  |
| Yes | 20 | | 10.8 | | 21 | | 6.3 |  |  |
| Antipsychotic intake**** |  | |  | |  | |  |  | 0.27 |
| No | 107 | | 57.8 | | 156 | | 46.7 |  |  |
| Yes | 78 | | 42.2 | | 178 | | 53.3 |  |  |
| Mood stabilizer intake**** |  | |  | |  | |  |  | 0.33 |
| No | 99 | | 53.5 | | 145 | | 43.4 |  |  |
| Yes | 86 | | 46.5 | | 189 | | 56.6 |  |  |
| Antalgic intake**** |  | |  | |  | |  |  | 0.95 |
| No | 164 | | 88.6 | | 294 | | 88.0 |  |  |
| Yes | 21 | | 11.4 | | 40 | | 12.0 |  |  |

Model 3: Adjusted for depression severity, age, sex, maximum psychological pain and lifetime SA; *not adjusted for lifetime SA (because these variables are only for patients with lifetime SA); **not adjusted for depression severity; *** not adjusted for maximum psychological pain

**** Classification according to the CIM-10: Psychotropics: N05; Anxiolytics/hypnotics: N05B and N05C; Antidepressants: N06A; Antiepileptics: N03A; Antipsychotics: N05A; Mood stabilizers: N03A and N05A; Antalgics: N02.

IDSC30: Clinician-rated 30-item Inventory Depression Symptomatology; QIDS: Self-rated Quick Inventory of Depressive Symptomatology; BDI: self-rated Beck Depression Inventory; RRRS: Risk/Rescue Rating Scale; SIS: Suicidal Intent Scale; BSSI: Beck Scale for Suicide Ideation; BIS: Barratt Impulsiveness Scale; BHS: Beck Hopelessness Scale; RFLI: Reasons for Living Inventory; AIM: Affective Intensity Measure; ALS: Affective Lability Scale; STAI: Anxiety using the State/Trait Anxiety Inventory; CTQ: Childhood Trauma Questionnaire (CTQ); VAS: visual analog scales (VAS); ESUL: University of California, Los Angeles Loneliness Scale; MARS: Medication Adherence Report Scale

Suppl Table 2: Risk of actual SA and SE (i.e. actual SA, aborted SA, interrupted SA, hospitalization for SI) during the 1-year of follow-up in patients with suicidal and non-suicidal depression

|  | Suicidal depression | | | | Model 2 | | Model 3 | |
| --- | --- | --- | --- | --- | --- | --- | --- | --- |
| Variables | No  N (%) | | Yes  N (%) | | OR [95% CI] | *p-value* | OR [95% CI] | *p-value* |
| **Actual SA** |  |  |  |  |  | **0.02** |  | **0.03** |
| No | 159 | 92.4 | 256 | 82.6 | 1 |  | 1 |  |
| Yes | 12 | 7.6 | 50 | 17.4 | 2.31 [1.15; 4.63] |  | 2.14 [1.06; 4.33] |  |
| **Suicidal event*** |  |  |  |  |  | **0.01** |  | **0.03** |
| No | 138 | 79.5 | 208 | 65 | 1 |  | 1 |  |
| Yes | 33 | 20.5 | 101 | 35 | 1.85 [1.15; 2.97] |  | 1.73 [1.07; 2.79] |  |
| **New actual SA (for patients with previous SA, N = 357)** |  |  |  |  |  | **0.01** |  |  |
| No | 81 | 93.5 | 170 | 79.2 | 1 |  |  |  |
| Yes | 5 | 6.5 | 41 | 20.8 | 3.73 [1.38; 10.09] |  |  |  |
| **New SE (for patients with previous SA, N = 357)** |  |  |  |  |  | **0.03** |  |  |
| No | 68 | 79.3 | 138 | 62.7 | 1 |  |  |  |
| Yes | 18 | 20.7 | 74 | 37.3 | 2.04 [1.09; 3.81] |  |  |  |
| **First actual SA (for patients without previous SA, N = 201)** |  |  |  |  |  | 0.79 |  |  |
| No | 78 | 91.4 | 84 | 90 |  |  |  |  |
| Yes | 7 | 8.6 | 9 | 10 |  |  |  |  |

Model 2: Adjusted for age, sex, baseline maximum psychological pain, and baseline depression severity
Model 3: Adjusted for age, sex, lifetime SA, marital status, professional activity, baseline maximum psychological pain, and baseline depression severity
*Suicidal event = actual SA, aborted SA, interrupted SA, hospitalization for SI

Suppl Table 3: Cox regression model to estimate the risk of actual SA during the 1-year follow-up in patients with suicidal and non-suicidal depression at baseline

|  | HR (95% CI) | *p-value* |
| --- | --- | --- |
| **Unadjusted model** |  |  |
| **Suicidal depression** |  | **0.001** |
| No | 1 |  |
| Yes | 2.43 (1.36 ; 4.36) |  |
| **Adjusted model** |  |  |
| **Suicidal depression** |  | **0.03** |
| No | 1 |  |
| Yes | 2.03 (1.06; 3.92) |  |
| **Age** | 0.97 (0.96; 0.99) | **0.008** |
| **Sex** |  | 0.81 |
| Men | 1 |  |
| Women | 1.07 (0.63; 1.82) |  |
| **Lifetime SA** |  | 0.15 |
| No | 1 |  |
| Yes | 1.54 (0.86; 2.73) |  |
| **Baseline depression severity** |  | 0.42 |
| Moderate | 1 |  |
| Severe | 1.25 (0.72; 2.17) |  |
| **Baseline maximum psychological pain** | 0.99 (0.84; 1.18) | 0.98 |

Suppl Table 4: Cox regression model to estimate the risk of SE (i.e. actual SA, aborted SA, interrupted SA, hospitalization for SI) during the 1-year follow-up in patients with suicidal and non-suicidal depression at baseline

|  | HR (95% CI) | *p-value* |
| --- | --- | --- |
| **Unadjusted model** |  |  |
| **Suicidal depression** |  | **0.001** |
| No | 1 |  |
| Yes | 1.98 (1.34; 2.92) |  |
| **Adjusted model** |  |  |
| **Suicidal depression** |  | **0.01** |
| No | 1 |  |
| Yes | 1.79 (1.15; 2.79) |  |
| **Age** | 0.98 (0.97; 0.99) | **0.002** |
| **Sex** |  | 0.62 |
| Men | 1 |  |
| Women | 1.10 (0.75; 1.61) |  |
| **Lifetime SA** |  | 0.17 |
| No | 1 |  |
| Yes | 1.32 (0.89; 1.96) |  |
| **Baseline depression severity** |  | 0.51 |
| Moderate | 1 |  |
| Severe | 0.88 (0.61; 1.28) |  |
| **Baseline maximum psychological pain** | 1.05 (0.93; 1.20) | 0.42 |
